# Stain-Free Quantification of Chromosomes in Live Cells Using Regularized Tomographic Phase Microscopy

**DOI:** 10.1371/journal.pone.0049502

**Published:** 2012-11-16

**Authors:** Yongjin Sung, Wonshik Choi, Niyom Lue, Ramachandra R. Dasari, Zahid Yaqoob

**Affiliations:** 1 G. R. Harrison Spectroscopy Laboratory, Massachusetts Institute of Technology, Cambridge, Massachusetts, United States of America; 2 Department of Physics, Korea University, Seoul, Republic of Korea; Glasgow University, United Kingdom

## Abstract

Refractive index imaging is a label-free technique that enables long-term monitoring of the internal structures and molecular composition in living cells with minimal perturbation. Existing tomographic methods for the refractive index imaging lack 3-D resolution and result in artifacts that prevent accurate refractive index quantification. To overcome these limitations without compromising the capability to observe a sample in its most native condition, we have developed a regularized tomographic phase microscope (RTPM) enabling accurate refractive index imaging of organelles inside intact cells. With the enhanced accuracy, we quantify the mass of chromosomes in intact living cells, and differentiate two human colon cancer lines, HT-29 and T84 cells, solely based on the non-aqueous (dry) mass of chromosomes. In addition, we demonstrate chromosomal imaging using a dual-wavelength RTPM, which shows its potential to determine the molecular composition of cellular organelles in live cells.

## Introduction

Refractive index has been used as a source of contrast in many optical imaging modalities such as phase contrast microscopy for routine observation of biological samples, and optical coherence tomography [1], [2] for *in vivo* cellular and tissue imaging. The refractive index is proportional to the concentration of organic molecules [3], [4], [5]; therefore, it has also been used for quantifying the aggregation [6] and surface coverage [7] of cellular proteins, and the growth [8], [9], [10] and architectural changes in cells [11], [12], [13]. Since refractive index imaging does not require external contrast agents or genetic manipulations [14], it can be easily applied to primary cells as well as established cell lines. In addition, the measurements can be accurately repeated without any concerns of the photobleaching or non-uniform binding of the labeling agents [15]. Recently, several groups [16], [17], [18] have demonstrated the feasibility of 3-D mapping of refractive index in live cells, promising the use of organelles’ refractive index as a biomarker to quantify the physiological status of cells.

However, the task of accurately determining the refractive index at the subcellular level is a challenging problem. Typically, light field scattered from a sample is measured at varying angles of illumination [17], [18], [19], [20], [21], [22], [23], [24], as in x-ray computed tomography, and a solution to the inverse problem is sought for the scattering of the incident light due to inhomogeneous refractive index distribution [25]. We note that the major obstacle in accurate measurement of refractive index is the incomplete collection of the scattered fields, mainly due to finite numerical aperture of the illumination and collection optics [17], [19], [20], [21], [22] or sample rotation around only one axis [18]. The incomplete sample information leads to serious distortions, known as the missing-angle artifacts [26], in the reconstructed refractive index map. Specifically, the reconstructed image is elongated along the direction of the optical axis or along the axis of sample rotation, and the value of measured refractive index is underestimated. More importantly, these effects strongly depend on the sample’s refractive index distribution itself. As a result, it has been difficult to accurately determine the refractive index of organelles in adherent metazoan cells, which are often amorphous and flexible in shape. One way to overcome this problem is to employ sample rotation in combination with beam scanning [23]. However, it will require placing the sample inside a holder, or optically tweezing it with highly focused beams. Either of these approaches is not ideal for long-term observation of live cells, hardly applicable to the cells growing adherent to a substrate.

Here, we report a regularized tomographic phase microscope (RTPM) that overcomes the above mentioned problems without additional measurements; it can, therefore, accurately measure the refractive index of organelles in live cells in their most native condition. To demonstrate this capability, we quantify the amount of non-aqueous content or dry mass of condensed chromosomes in the region where during metaphase the refractive index is distinguished from the cytoplasm by the presence of chromosomes. Solely based on the chromosomal dry mass, we show that HT-29 and T84 human colon cancer cells can be differentiated from each other. Furthermore, we show different amount of refractive index dispersion in chromosomes compared to the cytoplasm in HT-29 cells using a dual-wavelength RTPM, which may be further used to determine molecular composition of organelles in live cells.

## Results and Discussion

The interaction of light with an object can be represented by the scattering potential, which is the function of the object’s complex refractive index 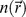:
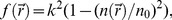
(1)where 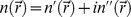; 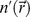 and 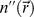 represent the phase delay and absorption, respectively of the light passing through the object. 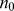 is the refractive index of the medium in which the sample is immersed. The scattering potential of a biological specimen can be determined by utilizing a series of measurements of the scattered field from the sample and solving an inverse problem that can be formulated as below:
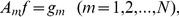
(2)where 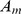 is the forward scattering operator, and 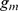 represents the measured scattered field. We note that both 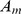 and 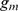 depend on the incident angle of illumination, and N is the number of measurements.

**Figure 1:**
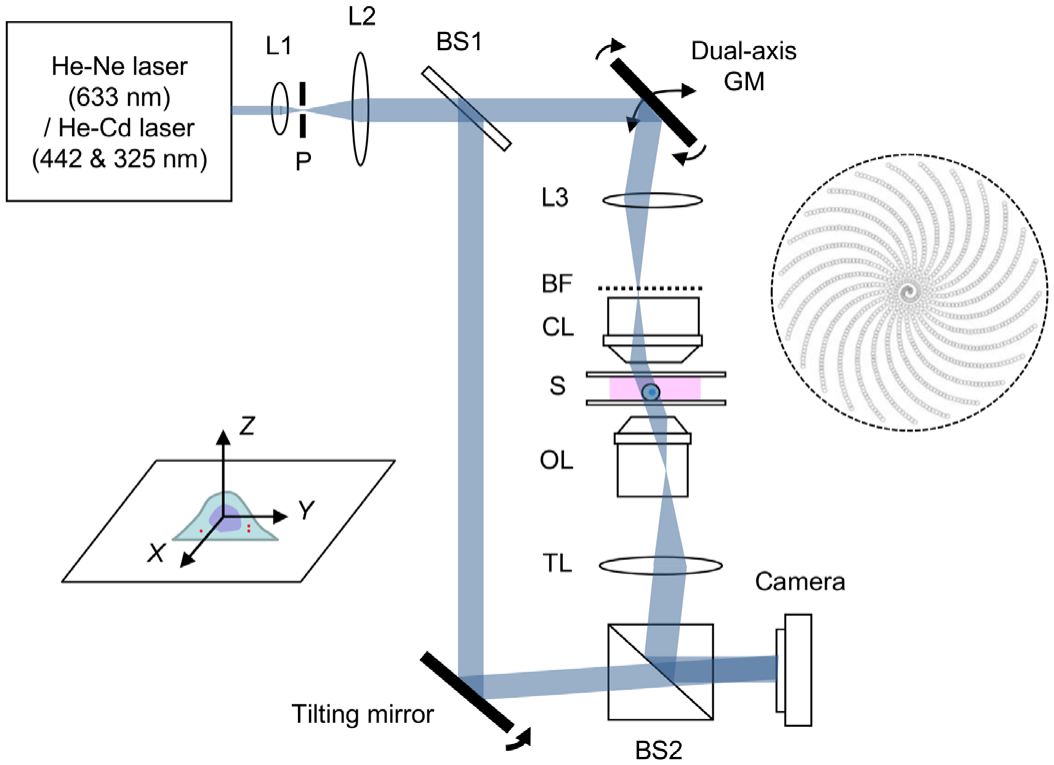
Schematic layout of the regularized tomographic phase microscope (RTPM) set-up. L: lens; P: pinhole; BF: back focal plane; CL: condenser lens; S: sample; OL: objective lens; TL: tube lens; GM: galvanometer mirror; BS: beam splitter. The open circles on the right represent a trace of the focused beam in the back focal plane of condenser lens when the angle of the incident beam is varied at the sample plane.

The scattered field 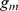 for a specific angle of illumination in Eq. (2) can be obtained from the intensity images recorded at two planes along the light propagation direction [27], [28], from a single intensity image and *a priori* information of the sample [29], [30], or using interferometry [31], [32]. In this study, we use a Mach-Zehnder-type interferometer [20], [32] for the scattered field measurement at varying angles of illumination (Fig. 1). The angle of the incident beam is varied by a dual-axis galvanometer scanner installed at the plane conjugate to the sample plane. In comparison with the angular scan along a line [17], [19], [20], [21] or two lines [22], the 2-D angular scan of the incident beam provides twice higher spatial resolution than the diffraction-limit in all the transverse directions [33]. We note that this 2-D angular scan is crucial for maximizing the spatial frequency support of the measured spectrum, and for reducing the ill-posedness of the inverse problem, Eq. (2). For each tomogram, we acquire 400 images within one second using a CMOS camera synchronized with the galvanometer scanner (see Materials and Methods for a detailed explanation).

Although the incident angle is varied in all the possible directions within the given numerical aperture of the illumination system, a significant portion of the 3-D object spectrum is still missing. However, the incomplete data collection can be compensated using *a priori* information of the sample in an iterative reconstruction framework [26]. Specifically, the following two constraints can be applied to the tomographic data acquired from biological cells: (i) positivity constraint and (ii) piecewise-smoothness constraint. The first constraint, which requires that the cell’s refractive index is higher than that of the culture medium, is justified because cells consist of densely packed solid materials and the refractive index is linearly proportional to their concentration. The use of the positivity constraint is similar to the idea of the Papoulis-Gerchberg algorithm that extrapolates a band-limited signal from only a part of the signal [34]. It is worth noting that the positivity constraint can be applied separately to real and imaginary parts of the complex refractive index [30]. The piecewise-smoothness constraint assumes that cell’s organelles have relatively smooth variation of refractive index inside each organelle, but steep gradient at the boundary. This is justified because the organelles of interest, e.g., nucleolus, lipids, condensed chromosomes, etc., usually have a refractive index distinct from that of cytosol and small details within the organelles are not either resolvable (due to the resolution limit) or of primary interest when the mean refractive index of an organelle is sought [35]. The piecewise-smoothness constraint can be incorporated as a penalty functional into the iterative algorithm, in which the sum of the penalty and fidelity terms is minimized for an optimum solution [36]. The use of the piecewise-smoothness constraint can be also connected to compressed sensing [37], [38]. It is worth noting that the suggested regularization algorithm is robust with noisy raw scattered field images since it diffuses noise while enhancing the boundaries [26].

**Figure 2:**
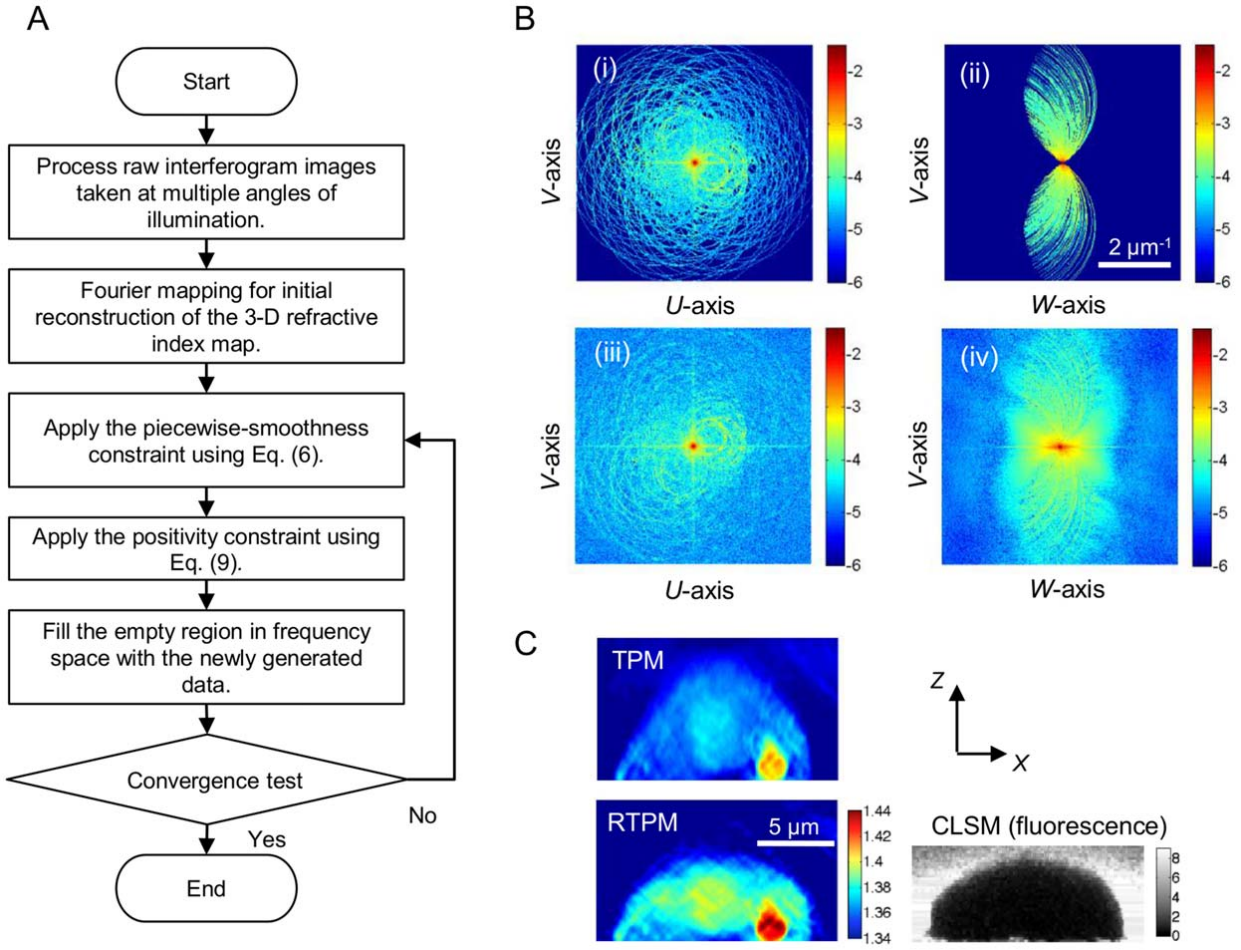
Data processing and validation. A: Flow chart of the iterative algorithm to retrieve the 3-D refractive index map in RTPM. B: Horizontal (i, iii) and vertical cross-sections (ii, iv) of the frequency spectrum of the 3-D refractive index maps before (i, ii) and after 50 iterations (iii, iv). The colorbars for images (i-iv) represent the amplitude of the frequency spectrum shown in logarithmic scale, base 10. C: Vertical cross-sections of the refractive index map for a HeLa cell obtained with TPM, RTPM, and CLSM. The value in the CLSM image is the amplitude of signal recorded using photomultiplier tubes, which represents the fluorescence intensity of fluorescein molecules added to the medium.

Figure 2A shows a flow chart of the proposed algorithm to process measured scattered fields and to retrieve the 3-D refractive index map [26]. First, the scattering potential is constructed from the measured scattered fields using an algorithm based on the Fourier diffraction theorem [19] (see Materials and Methods for details). This step provides a reasonable initial guess 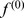 for the subsequent iterative process, and identifies the frequency support of the measured spectrum. During the iterative process, the scattering potential 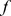 is updated by incorporating the new information generated from the additional constraints. Figure 2B shows an example of horizontal and vertical cross-sections of the frequency spectrum of a sample before (i, ii) and after (iii, iv) the regularization process. Initially, the frequency components are under-sampled at high spatial frequencies (i). Also, the missing cone region, resembling the shape of an apple-core, can be clearly seen near the origin of the coordinates in (ii). After regularization (50 iterations), the frequency space including the missing cone region is more uniformly filled (iii, iv). Figure 2C compares the vertical (or depth) cross-sections of a HeLa cell obtained using conventional and regularized tomographic phase microscopes. As shown, the cell is sitting on a 3-µm silica bead (see Materials and Methods for sample preparation). For sake of validation, we have installed a confocal laser scanning microscope (CLSM) to be used in conjunction with the tomographic phase measurements (see Methods S1 for more detailed description). We note that CLSM, when used with fluorescence labeling, is free from the missing-cone artifacts, and thus can be used to assess the RTPM’s capability to overcome the missing-cone problem in a conventional tomographic phase microscope (TPM). The height profile obtained with the RTPM is drastically different from that obtained with TPM, while it accurately matches with the CLSM measurement (Fig. 2C). Importantly, the measured refractive index value of the silica bead underneath the HeLa cell has changed from 1.405 to 1.425, which matches exactly with that obtained for single silica beads using index-matching method.

**Figure 3:**
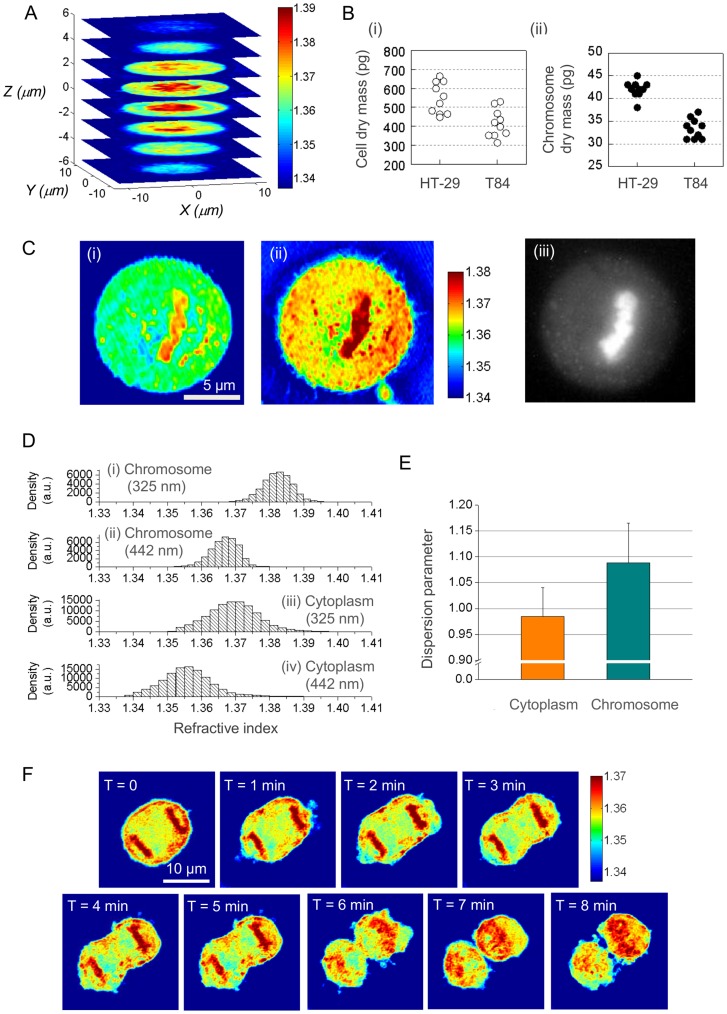
Quantification of chromosomes in live cells. A: Refractive index tomogram of a T84 cell in metaphase measured at 633 nm. B: Dry mass of the entire cell and that of the condensed chromosomes for HT-29 and T84 cells in metaphase. C: Dispersion of chromosomes in eukaryotic cells. Sample images of the refractive index map (cross-section) for a HeLa cell in metaphase measured at (i) 442 nm and (ii) 325 nm, respectively; (iii) a corresponding fluorescence image with nucleic acid stained with Syto13. D: Histogram of the refractive index for chromosomes in HT-29 cells (i, ii) and cytoplasm (iii, iv) at the wavelength of 325 nm (i, iii) and 442 nm (ii, iv), respectively. E: Dispersion parameter estimated from Fig. 3D and Eq. (4). The two distributions are statistically different (*p* = 0.0133). F: Label-free imaging of cytokinesis in a HeLa cell using RTPM. Cross-sections of the 3-D refractive index map are shown at different time points. Colorbars in A, C and F represent the refractive index.

Using RTPM, we have quantified condensed chromosomes in single HT-29 and T84 human colon cancer cells in mitotic phase. Figure 3A shows multiple horizontal cross-sections of the refractive index tomogram for a T84 cell, in which the chromosome region can be easily distinguished from the cytoplasm. From the linear relationship between the refractive index and the concentration of non-aqueous contents, the total chromosomal dry mass may be obtained from the integration of the refractive index map over the volume of chromosome region as below:
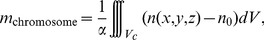
(3)where 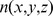 and 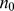 denote the refractive index (a dimensionless quantity) of the chromosome and the culture medium, respectively, while 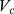 represents the chromosome region. The quantity 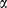 is the average specific refractive index increment, defined as the increase in refractive index value per unit increase in the concentration of the constituent molecules [39]. In this study, we adopt the value suggested by Barer [3], 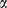 *= *0.18 (g/mL)^-1^.

Figure 3B shows dry mass of the entire cell in metaphase and that of the chromosomes for two human colon cancer lines, HT-29 (*n* = 10) and T84 cells (*n* = 10). The total dry mass of HT-29 cells (546.7±82.7 pg) is 32.0±27.3% larger than that of T84 cells (414.1±73.6 pg). On the other hand, the dry mass of the chromosomes in HT-29 cells (42.0±1.8 pg) is 25.8±8.6% larger than that of T84 cells (33.4±2.2 pg), which is in close agreement with the modal numbers obtained by cytogenetic analysis (http://www.atcc.org) for the two cell lines. The modal chromosome number for HT-29 cells (71) is about 26.8% larger than that for T84 cells (56). This agreement suggests that RTPM may be used to detect chromosomal abnormalities and to differentiate cell types in a label-free fashion.

In addition to the dry mass measurement, we have also determined the refractive index dispersion in order to quantify molecular composition at off-resonant wavelengths. Figure 3C shows the cross-section images of the refractive index maps recorded at (i) 442 nm and (ii) 325 nm for a HeLa cell in metaphase, which clearly shows differing amounts of dispersion for the chromosomes and the cell cytoplasm. For comparison, a wide-field fluorescence image of the same cell stained with Syto13 (S7575, Invitrogen) is obtained using a Zeiss microscope (with fluorescence imaging capability, and is shown in Fig. 3C (iii). Figure 3D compares the refractive index values for the chromosomes and cytoplasm in HT-29 cells at the two wavelengths: 1.367±0.004 (442 nm) and 1.382±0.004 (325 nm) for the chromosomes; and 1.356±0.009 (442 nm) and 1.369±0.008 (325 nm) for the cytoplasm. As a quantitative measure of the molecular composition, we define a dispersion parameter 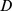 as.
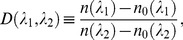
(4)where 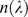 and 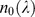 represent the refractive index of the sample and the surrounding medium, respectively, at the wavelength 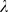. We note that optical absorption measurement at the deep-ultraviolet (UV) wavelengths has been recently demonstrated for stain-free mapping of nucleic acid and protein mass in a living cell [40]. For minimal UV-induced toxicity, however, it is preferable to use the refractive index contrast at off-resonant wavelengths.

Figure 3E compares the dispersion parameter averaged over the chromosome and cytoplasm regions in HT-29 cells. The dispersion parameter for the chromosome region was estimated as 1.088±0.076, whereas for the cytoplasm, it was found to be 0.984±0.056; the two distributions are statistically different (*p* = 0.0133, two-tailed *t*-test, *n* = 7). This difference is attributable to the difference in the dispersion of DNA and proteins. Figure 3F shows the cross-sections of the 3-D refractive index map of a HeLa cell going through cytokinesis. These images demonstrate the capability of RTPM to clearly illustrate and quantify cellular changes during cell division: segregated chromosomes near the pole of each daughter cell (0–5 min); asymmetric separation of cytoplasmic material into two daughter cells (6–8 min); and denaturing of chromosomes in the late stage of cytokinesis (6–8 min).

### Conclusion

In summary, we have reported a regularized tomographic phase microscope to provide accurate refractive index of cellular organelles. Specifically, we have: (i) adopted spiral scan for the maximum coverage of solid angle of the incident beam; (ii) applied the regularization algorithm to measured scattered fields for full 3-D reconstruction; and (iii) extended the regularized tomography approach for dispersion measurements. Without any staining, we have shown 3-D refractive index distribution of condensed chromosomes in HeLa, HT-29, and T84 cells. Both the HT-29 and T84 cells are human colon cancer cells, but their chromosomal dry mass values were measured to be significantly different, HT-29 cells (42.0±1.8 pg) and T84 cells (33.4±2.2 pg). This indicates that the reported method may be useful for label-free detection of cell ploidy. The study also demonstrates higher optical dispersion in chromosomes compared to cell cytoplasm, envisioning the potential of this technique to provide molecular composition of cellular organelles based on refractive index contrast.

## Materials and Methods

### Sample Preparation

HeLa, HT-29 and T84 cells were purchased from the American Tissue Culture Collection (ATCC), Manassas, VA, and cultured in Dulbecco modified Eagle medium (DMEM) supplemented with 10% FBS and 1% 100X penicillin-streptomycin solution. To study cells in metaphase, the cells were grown in a temperature-controlled flow chamber (Warner Instruments, RC-31) with continuous supply of conditioned media pre-equilibrated with air and 5% CO_2_. For the bead imaging underneath a cell (Fig. 2C), we coated a coverslip with poly-L-lysine (Sigma-Aldrich, P8920), and dispersed diluted 3-µm silica beads (24330-15, Polysciences, Inc.) on the surface of the coverslip. After completely drying the coverslip, 2–3 drops of media containing suspended cells were carefully placed on the coverslip. After incubating the sample for a couple of hours, we could occasionally find cells sitting on a bead. Finally, we added fluorescein, which has slow rate of uptake by cells, to obtain the cell boundary as a negative of the fluorescence signal when imaged using a CLSM. For the fluorescence image in Fig. 3C(iii), we stained the cells with Syto13 (S7575, Invitrogen) following the protocol provided by the manufacturer.

### Experimental Set-up

Figure 1 shows the schematic layout of the RTPM set-up (see Fig. S1 for a complete layout including the CLSM set-up). A Mach-Zehnder-type interferometer, where a reference beam is combined with the beam passing through a sample, is used to measure the scattered field at different angles of illumination. A dual-axis galvanometer scanner (Model 6650, Cambridge Technology) is installed at a plane conjugate to the sample plane to vary the illumination angle on the sample. The open dots in Fig. 1 represent a trace of the focused beam in the back focal plane of the condenser lens while the illumination angle is varied; each point represents a collimated beam incident onto the specimen with a specific angle of illumination. As illumination source, we used a He-Ne laser (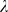 = 633 nm) to measure the mass of condensed chromosomes, and a He-Cd laser (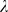 = 442 nm and 325 nm) to measure their dispersion. A complementary metal oxide semiconductor (CMOS) camera (1024PCI, Photron) was triggered to capture 400 images of scattered field (for different angles of illumination) in less than a second. UV grade high-NA objective lenses were used as the condenser (1.25 NA, Partec) and imaging (Fluar 1.3 NA, Zeiss) lenses.

### Initial Reconstruction of the 3-D Refractive Index Map

With the first Rytov approximation, which is valid for most biological samples [19], the field scattered from an object can be connected to the object’s spatial frequency spectrum as below [19], [41]:
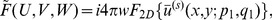
(5)where 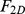 represents the 2-D Fourier transform, and 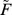 is the 3-D Fourier transform of the scattering potential 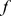. The function 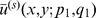 is the scattered field measured for the incident beam with the wave vector 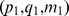, where 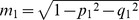. The variables 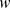 and 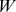 are defined as 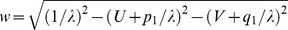 and 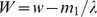, respectively. For each illumination angle, the scattered fields are mapped to different locations in the 3-D spatial frequency space (Fig. S2); therefore, different portions of the object spectrum can be obtained by the angular scan of the incident beam. The sample’s scattering potential, and thus 3-D refractive index map can be obtained by taking the inverse Fourier transform of this mapping.

### Iterative Reconstruction using *a priori* Knowledge

After the initial reconstruction, the function 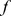 is updated in the following iterative process:
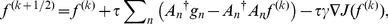
(6)where 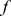 is the objective function, and the superscript 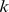 indicates the iteration number. 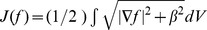 is the penalty functional, where 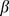 is an arbitrary small number that prevents 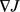 from having a singular value for 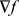 = 0. The variable 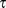 is a relaxation parameter that determines the speed of convergence, and 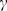 is a regularization parameter that represents a trade-off between the data fidelity and penalty terms. The regularization parameter (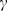 = 10^-5^) was chosen using a numerical analysis on the sample with a refractive index value similar to real biological samples. We also checked that the accuracy of reconstruction was not sensitive to the choice of the regularization parameter (Fig. S3). Furthermore, 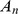 and 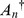 are the forward and adjoint operators defined by:



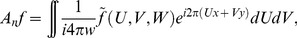
(7)

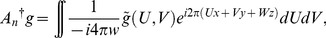
(8)where 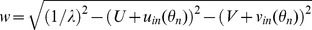 and 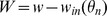. The variables 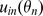 and 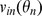 are 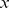 and 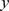 components of the incident wave vector, and 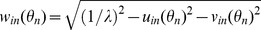.

The positivity constraint can be incorporated into the iterative algorithm as follows [36].
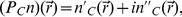
(9)where



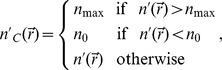


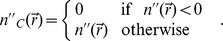


The data generated from these constraints are used to fill only the empty region, where the measurement data are not available.
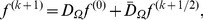
(10)where 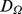 is the band-limiting operator preserving only the components inside the frequency band 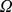; 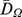 is a complementary operator to 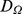 preserving only the components outside of the frequency band; and 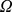 is the frequency support of the measured spectrum.

The iteration is continued until the following criteria for convergence is attained:
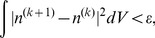
(11)where 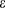 is a small number that decides the iteration error.

## Supporting Information

Figure S1 Schematic layout of the experimental set-up (TPM & CLSM) and performance test of CLSM.
(PDF)

Figure S2 Retrieval of scattered fields and 3-D mapping based on the Fourier diffraction theorem.
(PDF)

Figure S3 Sensitivity analysis of the regularization parameter.
(PDF)

Methods S1 Confocal laser scanning microscope (CLSM), and Retrieval of complex scattered fields from measured interferogram images.
(PDF)
