# Supplementary material for: Stain-Free Quantification of Chromosomes in Live Cells Using Regularized Tomographic Phase Microscopy
Source: PLoS One. 2012 Nov 16;7(11):e49502. doi: 10.1371/journal.pone.0049502 (PMC3500303; doi:10.1371/journal.pone.0049502)
Supplement: Figure S1 — Schematic layout of the experimental set-up (TPM & CLSM) and performance test of CLSM. (PDF) [file pone.0049502.s001.pdf]

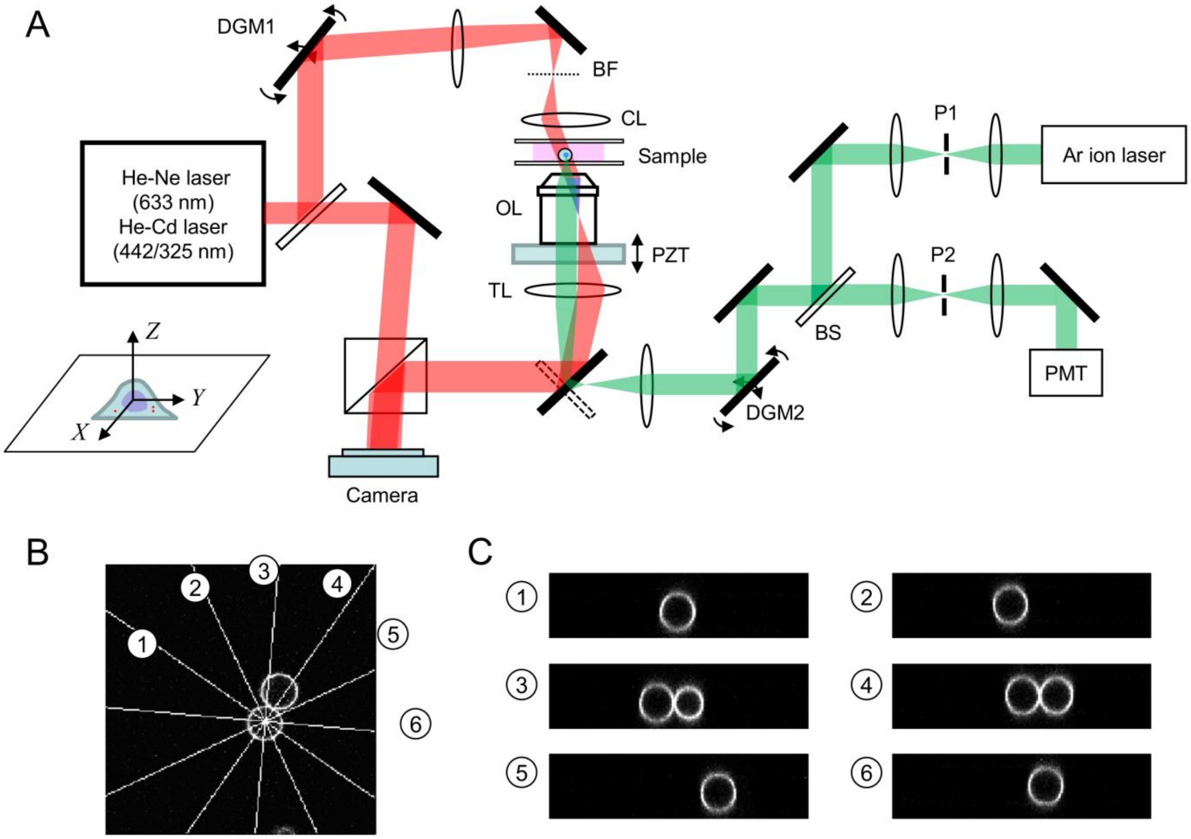

Figure S1. Schematic layout of the experimental set-up (TPM & CLSM) and performance test of CLSM. A: Schematic layout of the RTPM (red) and CLSM (green) set-ups shown together. P: pinhole; BF: back focal plane; CL: condenser lens; S: sample; OL: objective lens; TL: tube lens; DGM: dual-axis galvanometer mirror; PZT: piezoelectric transducer; BS: beam splitter; PMT: photo-multiplier tubes. B: A horizontal cross-section of two adjacent 6 μm diameter FocalCheck microspheres (F-14808, Invitrogen), acquired with the confocal laser scanning microscope (CLSM). C: Vertical cross-sections acquired using the CLSM along the lines in B.
