# Supplementary material for: Stain-Free Quantification of Chromosomes in Live Cells Using Regularized Tomographic Phase Microscopy
Source: PLoS One. 2012 Nov 16;7(11):e49502. doi: 10.1371/journal.pone.0049502 (PMC3500303; doi:10.1371/journal.pone.0049502)
Supplement: Figure S2 — Retrieval of scattered fields and 3-D mapping based on the Fourier diffraction theorem. (PDF) [file pone.0049502.s002.pdf]

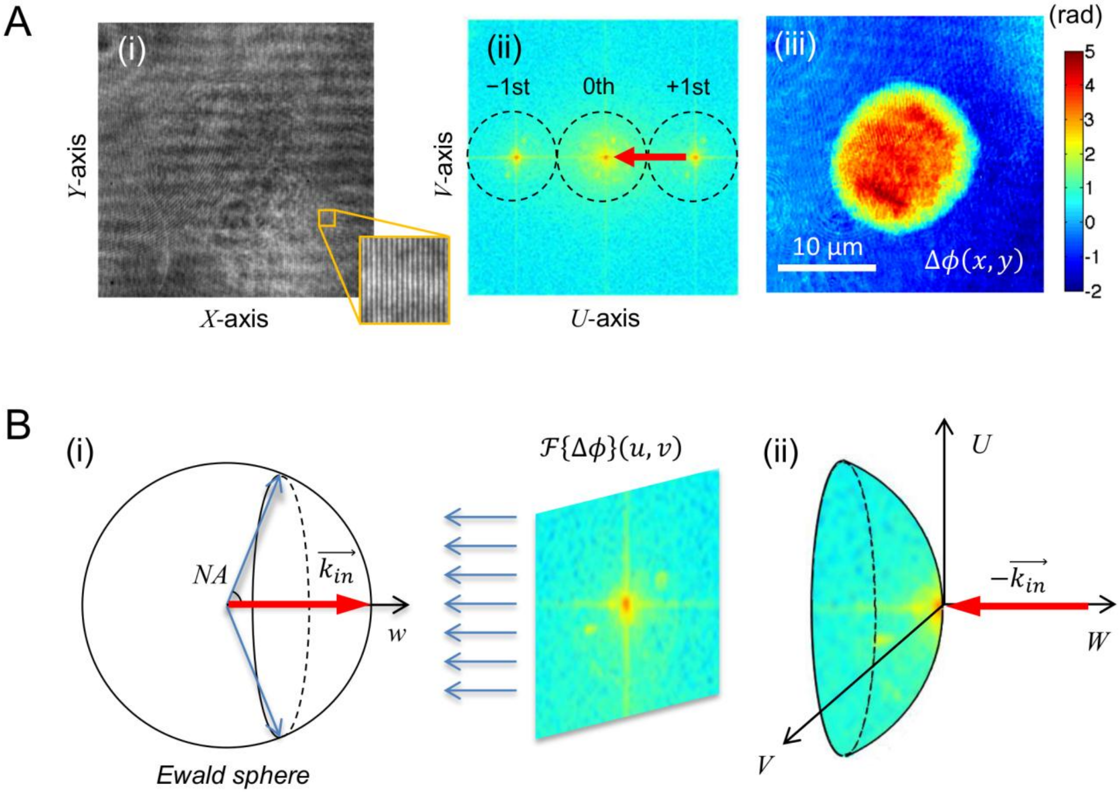

Figure S2. Retrieval of scattered fields and 3-D mapping based on the Fourier diffraction theorem. A: Data processing to retrieve the sample-induced phase delay or phase image: (i) raw (or measured) interferogram; and (ii) the amplitude of Fourier transform of (i) shown in logarithmic scale, base 10. In (ii), the region inside the circle around either +1st- or -1st-order component is selected and shifted to the origin of the frequency coordinates. The inverse Fourier transform of the resulting spectra provides the phase image as shown in (iii). B: Example illustrating the mapping of measured scattered fields onto the Ewald sphere to provide the sample's spatial frequency spectrum.
