# Supplementary material for: Stain-Free Quantification of Chromosomes in Live Cells Using Regularized Tomographic Phase Microscopy
Source: PLoS One. 2012 Nov 16;7(11):e49502. doi: 10.1371/journal.pone.0049502 (PMC3500303; doi:10.1371/journal.pone.0049502)
Supplement: Figure S3 — Sensitivity analysis of the regularization parameter. (PDF) [file pone.0049502.s003.pdf]

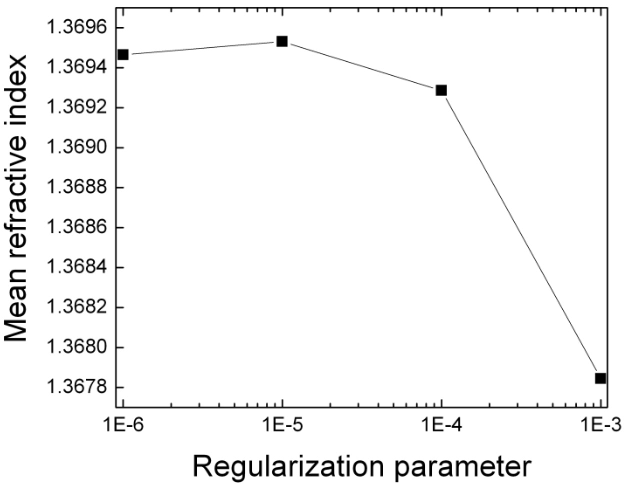


**Figure S3. Sensitivity analysis of the regularization parameter.** The regularization parameter $\gamma$ in Eq. (6) determines the trade-off between the penalty term and the fidelity term. Here, we use a numerical simulation to check how the choice of the regularization parameter affects the quality of reconstruction. Specifically, we compare the mean values of refractive index maps reconstructed for different values of the regularization parameter. The sample is a homogeneous spherical bead with refractive index 1.37 and diameter 8 $\mu$m. The number of phase images used for the reconstruction of one tomogram is 200 whereas the maximum angular coverage of the incident beam is $60^o$ with respect to the optical axis. As Fig. S3 shows, the mean refractive index value does not change much even if $\gamma$ is changed by two orders of magnitude from $10^{-6}$ to $10^{-4}$. However, if $\gamma$ is increased further to $10^{-3}$, the penalty term overwhelms the data fidelity term, leading to over-smoothing of the refractive index map and thus underestimation of the refractive index value itself.
