# Supplementary material for: Stain-Free Quantification of Chromosomes in Live Cells Using Regularized Tomographic Phase Microscopy
Source: PLoS One. 2012 Nov 16;7(11):e49502. doi: 10.1371/journal.pone.0049502 (PMC3500303; doi:10.1371/journal.pone.0049502)
Supplement: Methods S1 — Confocal laser scanning microscope (CLSM), and Retrieval of complex scattered fields from measured interferogram images. (PDF) [file pone.0049502.s004.pdf]

Supplementary Methods S1

Confocal laser scanning microscope (CLSM)

Figure S1 shows the experimental set-up including the confocal laser scanning microscope (CLSM) that is omitted in Fig. 1 for brevity. Both the TPM and CLSM set-ups are built on a Zeiss Observer.D1 microscope that has two video ports, so the TPM and CLSM measurements can be easily applied to a same specimen. A focused beam from an Ar-ion laser (λ = 488nm) is scanned in the transverse and axial directions across a sample using a dual-axis galvanometer scanner (Model 6732, Cambridge Technology) and a PZT-mounted microscope objective scanner (P-725, PI), respectively. The intensity of the backscattered light is measured by a photomultiplier tube (R6357, Hamamatsu), an amplifier (C7319, Hamamatsu), and a data acquisition board (PCI-6251) at the rate of 100 kHz. MATLAB (Mathworks Inc.) is used for the control of the TPM and confocal reflectance set-ups, data acquisition, and data processing.

Retrieval of complex scattered fields from measured interferogram images [1,2]

Figure S1 describes data processing to retrieve the scattered field, both the amplitude and phase, from a recorded interferogram image as shown in (i). The recorded image is the interferogram between the beam propagating through the sample and the reference beam through the free space. The latter was slightly tilted with respect to the former to create a spatial fringe. The irradiance in the image plane can be written as:

$$I(x,y) = I_S(x,y) + I_R + 2\sqrt{I_S(x,y)I_R}cos\big(2\pi qx - \Delta\phi(x,y)\big), \qquad [S1]$$

where $I_S(x,y)$ and I_R are the irradiances of the sample and reference beams, respectively, and $\Delta\phi(x,y)$ is the phase difference between the two beams. q the spatial frequency of the fringes in the raw interferogram image defined by the angle between the sample and reference beams in the detector plane. The image (ii) shows the magnitude of the Fourier transform of Eq. (S1) on a logarithmic scale (base 10). Three dotted regions around the peaks represent the 0th-order (center), and ±1st-order components (left and right), respectively. The radius of the circles indicates the numerical aperture of the imaging system. The +1st-order component can be written as

$$\hat{I}(u,v) = \iint \sqrt{I_S(x,y)I_R}e^{i\left(2\pi qx - \Delta\phi(x,y)\right)}e^{-i2\pi(ux+vy)}dxdy = \widehat{\Phi}(u-q,v), \qquad [S2]$$

where $\widehat{\Phi}(u, v)$ is the Fourier transform of $\Phi(x, y) = \sqrt{I_S(x, y)I_R}e^{-i\Delta\phi(x,y)}$. Note that the +1st-order and −1st-order components contain exactly same information of the sample. The function $\Phi(x, y)$ can be easily obtained by selecting only the region in the dotted circle around the +1st-order peak, moving it to the origin of the frequency coordinates, and taking its inverse Fourier transform. The image (iii) is the phase image obtained from the raw interferogram (i). The interferogram images recorded at varying angles of the incident beam are similarly processed and used for the 3-D reconstruction of refractive index map as explained below.

Supplementary References

1. Ikeda T, Popescu G, Dasari RR, Feld MS (2005) Hilbert phase microscopy for investigating fast dynamics in transparent systems. Optics letters 30: 1165-1167.
2. Sung Y, Choi W, Fang-Yen C, Badizadegan K, Dasari R, et al. (2009) Optical diffraction tomography for high resolution live cell imaging. Optics express 17: 266-277.
